# A Review and Integrative Analysis of Ancient Holistic Character Medicine Systems

**DOI:** 10.1100/tsw.2007.272

**Published:** 2007-11-12

**Authors:** Søren Ventegodt, Suzette Thegler, Tove Andreasen, Flemming Struve, Susan Jacobsen, Margrethe Torp, Hans Ægedius, Lars Enevoldsen, Joav Merrick

**Affiliations:** ^1^ Quality of Life Research Center, DK-1452 Copenhagen K, Denmark; ^2^ Research Clinic for Holistic Medicine, Copenhagen, Denmark; ^3^ Nordic School of Holistic Medicine, Copenhagen, Denmark; ^4^ Scandinavian Foundation for Holistic Medicine, Sandvika, Norway; ^5^ Interuniversity College, Graz, Austria; ^6^ National Institute of Child Health and Human Development, Jerusalem, Israel; ^7^ Office of the Medical Director, Division for Mental Retardation, Ministry of Social Affairs, Jerusalem, Israel; ^8^ Kentucky Children's Hospital, University of Kentucky, Lexington, KY, USA

**Keywords:** clinical holistic medicine, character medicine, Hippocrates, Ayurveda, Yin-Yang, cosmology, salutogenesis, energy-medicine, CAM, mind-body medicine, sense of coherence

## Abstract

The ancient holistic medical systems help the patient by balancing the “elements” of the human character. This work aims to understand the nature of these elements and the process of the physician balancing them. Using the concept of poly-ray cosmology we see that the medical systems from ancient India, China and Greek basically share the same inner structure and also the same logic of the treatment processes. We analyze the double concept of yin-yang, the Ayurvedic triadic concepts of Pitta, Kapha and Vata, the four elements of Hippocratic humoral medicine, and the five elements of Chinese medicine, and find that each of these conceptual frameworks make up a “theory” or model of the world that is a perfect wholeness, allowing the physician to interpret the world and his patient in order to identify the imbalances of his or her character that need to be treated. Independently of the system this can be a palliative treatment, if energies are only balanced in present time, or a causal cure if the physician is using the similarity principle to take his patient into regression back to the events in the personal history that originally created the imbalances (the traumas). To help the patient back to the traumas he is exposed to a small dose of the original harming stimulus; this can be an internal process like visualization supported by the therapist words, or an external process provoked by his actions. If the physician is balancing the elements without such a healing of the patient's existential core this can still momentarily help the patient by alleviating the symptoms, but it will not have a permanent effect.

### INTRODUCTION

In the emerging science of integrative medicine there has been a search for a uniform way to describe the different ancient holistic medical systems like the famous, original Hippocratic medicine[1], and to understand the way they work. The "character medicine" comes in many different forms, but seems always to heal the patient through “balancing the elements of the patient’s character”[2-6], but the nature of these systems and the way they cure patients have been difficult to explain. It seems that the treatment is basically interfering with the patient’s consciousness in subtle ways, helping him or her to regain faith, love, strength etc. Character is known to be closely related to human resources through the concepts of quality of life, coherence and life mission: the person who steps into character is using his ability and engaging his true self in his life-endeavours[7-14].

The search for an integrative system including many different treatment-systems have been ongoing, and Kratky[15] inspired by historical findings of such a solution in old Chinese texts, suggested that a six-element system can integrate all the above mentioned systems. The search by a culture for a deeper understand of the universe by increasing the abstract number lying at the root of the cultures cosmology seems to be a classical phenomena in the development of deep and spiritual cultures, very much the same way as materialistic cultures has limited the complexity of the cosmology to make the culture efficient, businesslike, and fast.

The development of worldviews – both towards complexity and simplicity - has always been problematic, as world-view seems to come in paradigms. We thus believe that each classical treatment system is based on a certain cosmology with a well-defined level of complexity embedded in the rituals of the culture. Often such a cosmology leads to a complete and closed interpretation of the world that cannot be expanded just by adding more elements. Tibetan medicine is an example of an integrative “meta”-medical system that has incorporated most of the cosmologies mentioned in this paper.

The difficulty of expanding a cosmological system with more elements is seen by the transformation of the significance of concept of “fire”. “Fire” appears as an element both in the ayurvedic, the Hippocratic and the Chinese cosmology, but the meaning of the concept “fire” is not identical, but not so different either. Kratky strived to create an overview of different cosmologies and of the consequences of these cosmologies for an approach to complementary medicine. Kratky enhanced common features of different cosmologies. On could argue that Kratky ignored the autonomy of each cosmology by the way he introduced “flora”, but from a pedagogical point of view, this is clearly profitable.

We are strong believers in the Antonovsky concept of salutogenesis (Aaron Antonovsky, 1923-1994) and when we try to understand the different medical systems we use the idea of such a healing of human existence suggested by Antonovsky.

### METHODS

In this paper we analyse the different ancient medical systems by using the concept of poly-ray cosmology[13]. The world can be described by such a cosmology in a way that covers all aspects of it; and any element in the world will be seen as carrying the fundamental characters that is dictated by the root structure of the cosmology. This backbone is characterised by the number of rays that the cosmology uses, which is revealed by the number of fundamental elements that is present in the description of the world. The number of elements can be one to many; a one-ray cosmology is monistic and typically materialistic (i.e. “all there exist is atoms”); a two-ray cosmology is dualistic like the Decartian dualism between mind and matter. The cosmology of ancient Greek containing the Hippocratic humoral medical system is a 4-ray cosmology with four fundamental elements, four types of human personality etc. The cosmologies of old India and old China are complex poly-ray cosmologies, which are only seemingly two- or three ray cosmologies; in that the fundamental concepts are really hiding an unexpected complexity**.**

After describing these elements we derive simple medical systems from the condensed “theories”, and demonstrate casuistically that they actually work in clinical practise.

### RESULTS

What we found was that there are simple cosmological schemes for the ancient holistic-medical systems that easily can be used to analyse, diagnose and treat a patient (see table 1).

**Table 1.**
**Four Different Holistic Medical Systems Based on Different Numbers of Elements (the basis of four types of "character medicine", see text.)**

| 2 elements | | Ayurveda – 3 elements | | | Hippocratic - 4 elements | | | | Chinese - 5 elements | | | | |
|---|---|---|---|---|---|---|---|---|---|---|---|---|---|
| Yang | Yin | Pitta | Kapha | Vata | Earth | Water | Air | Fire | Water | Wood | Fire | Earth | Metal |
| Hot Dry Repletion External Light Above Up Masculine | Cold Wet Vacuity Internal Dark Below Down Feminine | Oily Spicy Hot Light Pungent Mobile Liquid | Wet Cold Heavy Dull Slimy Soft Static | Dry Light Cold Rough Subtle Propelling | Firm Heavy Steady Dark Hard Feed Protect | Floating Dynamic Emotion Persevering Feminine | Easy Inspiration Light Spirit Communication | Transformation Warm Destructive Masculine Creative | Salty Winter Cold Death Black Fear Bones Ears | Rage Sour Spring Wind Birth Green | Joy Growth Heat Summer Red Bitter | Worry Sweet Change Yellow Dampness Late summer Transition | Sadness Spicy Fall Regression White |

### The Developmental History of Cosmologies in Cultures and Medical Systems

It is important to stress that the real complexity of the use and development of the medical systems in the different cultures are not the issue for this paper. The cosmologies have changed over time both in Greece, India, and China. In Hippocratic times, there where medical systems of six Chinese elements – thus 6 are believed by some researchers to be the original number. Later the number changed to 5 – which can be found in traditional Chinese medicine. By contrast, the number of Greek elements increased. There has been a shift from three elements (without earth) to four and later to five elements (including ether). The version of 4 elements was incorporated in Hippocratic medicine. Interestingly enough, the 3 doshas and 5 elements of Ayurveda are closely connected with the 3 (earlier) and the 5 (later) Greek elements, respectively.

The three doshas are said to correspond to the elements water, fire and air, but there is also 5 Ayurvedic elements, on the other hand, that have practically the same names as the 5 later Greek elements. These Ayurvedic elements are not discussed below. It is hard to tell for us to what extent the ancient cultures exchanges information, and it might be that all cultures actually have all cosmologies as subcultures, and that the information that we have gathered only is the information kept by historians because of its dominance in the actual culture. The point we try to make in this paper is not dependent of the historical development or cultural interchange; please forgive us for simplifying things to make them comprehensible.

### YIN AND YANG

The Yin-Yang principle represents a complete worldview. They do only exist as a pair, nor as opposite to each other but as elements that completes and balances each other equally. Yin contains an element of Yang and vice versa.

Yin and Yang are a symbolic concept. Each of them contains a rank of descriptive and explanatory elements witch characterise and together deepens the understanding of Yin-Yang (see table 1). Yin and Yang is a cyclic concept, which describe the movement and changes of life and nature, the order of "everything" e.g. the movement from light to dark and from day to night and so on. Seen through the glasses of Yin-Yang our complex world becomes immediately accessible and relative easily understandable. The ill person is seen as a person with unbalance in Yin-Yang. The treatment concerns revealing the patients unbalance and to address the unbalance in different ways e.g. with acupuncture or herb medicine in order to balance the person and create harmony. It can be argued that the Yin-Yang principle is part of traditional Chinese medicine; thus the Yin-Yang worldview is not as complete as suggested above.

### AYURVEDA AND THE THREE DOSHAS

The 3 basic elements – doshas – in Ayurvedic medicine are called Kapha, Pitta and Vata (see table 1). They are said to correspond to the elements water, fire and air[15], but this could be disputed. To these three elements certain characteristics are connected, which could also be called "humours"or "energies". If one of these characteristics is dominating an imbalance will occur. In our interpretation there are two levels on which the imbalances can arise [see also 15]:

- On the level of one's character (imbalance of the humours/energies)
- On the level of one's destiny (imbalance on a deep existential level)

The triad of Kapha, Pitta and Vata can be seen as elements, which coexist in a complementary way, you could say that they represent a circle of life. Kapha is what holds things together; Pitta digest things and Vata moves things. In another expression Kapha is the created, the earth, Pitta is the destructive fire and Vata is the creating element in life, symbolized by air.

In our interpretation these three forces can be taken to yet another level of mind, body and spirit. In this sense Vata is to be seen as mental understanding (movement), Pitta stands for the metabolic processes in the body (digestion) and Kapha for elements like love and compassion (holding things together). This view can perhaps be further understood by looking at the Ayurvedic perspective illnesses represented in the three doshas: Vata is connected to ailments and states of distress, Pitta to acute diseases and Kapha to chronic illnesses. A mixture of the three doshas characterizes a balanced person. But a mix of 2 doshas dominates most people and thereby a two-dosha system must be taken into consideration.

### Hippocrates' Humoral Medicine Based on the Four Elements

The kind of medicine we know in the west took, like other sciences, it's beginning in a religious milieu. The Greek treatment of patients in caves and temples sacred to Asklepios was based on dreams the patient had during his/her visit. The focus on knowledge of the Classical Era brought an increasing interest in scientific perspective on illness and health. Hippocrates (460-377 BCE)[1] who operated in a Asklepios shrine posed a theory on four bodily fluids ideally circulating the body unblocked. Blockings a certain spot gave way to unbalance and finally illness. The four elements water, air, fire and earth correspond in this system with a series of qualities from human and cosmos – being psychological (from psyche=soul) as well as bodily and natural states. The correspondences of the elements were developed in late antiquity.

That we here speak of a cosmology, which is inseparable from consciousness, is even clearer, when we look at the method of treatment. In the words of Hippocrates, that "the same [or the similar] cures the same" (similia simillibus curentur)[1] gave opportunity to treat by bringing the patient back to the cause or situation that originally made him/her ill. This is in a way the same procedure as in rituals.

The fact that the Hippocratic basic principles have survived relatively unaltered over more than 2000 years, tells us that the foundation should not be seen as fallible physiological or material knowledge, but as medicine of consciousness. Traditional Hippocratic medicine with cures like pelvic massage for hysteria is no longer used by European physicians, but reconditioned versions are used worldwide, i.e. Hahnemann's homeopathy counting for 10% of all medical consultations worldwide, and the much less known system of Unani or Yunani practiced in some Asian countries including the Islamic regions of India.

Human being as a religious creature is not separated from cosmos (the world), but connected through consciousness. The Greek conception, which serves this viewpoint, is to be understood from a holistic perspective, where soul, body and cosmos, the Great Whole if you prefer, are distributed in four partitions. Nothing is missing, or, every lack is to be found inside the system, in an unbalance or blocking of the flow/current. Interestingly one state can be transformed to another or a new state, which seems close to the way rituals work – in initiations or in Asclepiads – as a procedure. Here a model of four elements is applicable, since it is simple. The original situation is called into the light and transformed into a new existential state of the patient.

### Chinese Medicine and The Five Elements

In Chinese medicine the five element theory plays an important and central role. This system is a sort of interpretation of the universe that comprehends all existing, inclusive the human being. The five elements are wood, fire, earth, metal and water. Every single element has its own qualities and characteristics. For example a certain organ, colour, season, taste, emotion, smell, bodily condition, and pair of meridians. All elements are connected in a system, where they depend on and influence each other through nourishing and control. The energy flows through our body and the meridians in an ever-circulating movement. When the energy flow is harmonious we feel well and healthy. When the energy is disharmonious we will experience deficiency and illness.

In the clinic we can list the symptoms of the patients according to the elements, by asking, listening and observing. And finally we will get a picture of which elements needs balancing. In the West we are about to accept acupuncture as a pain treatment, but to get the full value of this system and be able to use it as a holistic system, we first have to understand the deep Eastern philosophy behind it.

### A Cosmology Can Contain Many Perspectives As The Elements Can Rotate

In trying to understand the nature of the tree key concepts of Ayurveda we tried to create a tree-ray cosmology, using the three fundamental Indian characters: Creation, maintenance, and destruction. We were able to attribute a number of qualities to these three key elements:

- Creation: coherence, fantasy, birth, sex, giving, surplus, power, realisation, love
- Maintenance: preserving, protecting, security, firmness, helpfulness, reality, order, strength
- Destruction: Fun, trouble, cleansing, renewing, transformation, anxiety, magic, death, spice, wildness, devil, chaos, anarchy, analysis

These elements were very different from the three basic element of Ayurveda, but when a new triangle was made, which covered the intermediate positions (between "Creation" and "Maintenance" etc.), the qualities of Pitta, Kapha and Vata (see table 1) emerged. Therefore it was possible to understand these three elements though a simple transformation into another perspective.

### CASE REPORT 1

#### Treating John with Yin-Yang Balancing [patient's name has been changed]

To help John to be an upgraded version of himself, the therapist (a volunteer from the group) made a diagnostic analysis of his yin and yang profile. The therapist found that John had too much yin and too much yang also. As the qualities related to yin were definitely different from the qualities related to yang this was not theoretically impossible, but still somewhat surprising. John reflected on the meaning of this and found that he in general needed to engage more in life and social life, not to accumulate the energy, but to permit it to flow allowing him to be more useful and more a part of the world. By balancing his yin and yang the therapist helped him use himself better. As he did not go back in time, when his negative patterns were established, his treatment was not a causal cure but a treatment of his symptoms – his "phlegmatic" personality. The treatment did not include other procedures.

Please notice that within the simple version of presenting Yin and Yang in table 1 as just complementary elements, however, the case of John would be impossible.

### CASE REPORT 2

#### Treating Beatrice With Acupressure [patient's name has been changed]

Beatrice was found to have too much "water" and to little "fire", when evaluated by the therapist (a volunteer from the group) using the five-element-scheme. She was negative, black, dead and cold, like winter, and needed to cheer up and be more joyful, growing and warm like the summer. The bladder meridian was weakened and the heart meridian was strengthened, and she felt very well and happy indeed after the session, where she travelled all the way back to childhood and the original establishment of her sad, heavy and quite depressive personality. This treatment was thus a causal treatment for her depression. By intending the shift the therapist send Beatrice into deep regression, but only because Beatrice herself intended the healing to happen. The treatment did not include other procedures.

### What is the Use of the Systems?

All the four systems we have went through, Yin and Yong – Ayurveda - the Western four element system and the Chinese five element system, can be used for both diagnosing as well as treatment of unbalance in patients.

How is the original reason found? It is very interesting to notice the momentarily improvement of a patient, who after a long search finally finds and understands the existential reason for an illness. Induction of spontaneous healing follows the appearance of a clear and certain sense of being in healing and coherence.

How does the treatment change when the reason has been found? The focus will now change from introspective work to the outer world: how to lead a good and healthy, positive, productive, and constructive life in the future.

### The Diagnosis

Diagnostics is carried out by very carefully looking at the separate elements in the chosen system, identifying this element in the patient’s character (in the body, consciousness, “energy”, “meridians, “personality”, or “energetic body/aura”) together with the sub elements, which forms the single elements for example cold, joy, rage etc. Very carefully observing and interviewing the patient allows the holistic therapist to give the patient a diagnosis. The unbalanced element(s), where the patient has to much or too little “energy” are thus determined.

Diagnosis is based on the physician’s interpretation of the patient unbalance in character as materialised by symptoms from spirit, mind, emotions, or body. Diagnosis thus depends on intuition and a very good mutual emotional contact and confidence between the patient and the therapist. This is very different from contemporary biomedicine.

### The Treatment

When the unbalances are traced, the treatment can be carried out by adding or subtracting, or moving “energy” from the elements. Treating the patient is also based on mutual confidence and emotional contact between the therapist and the patient and can in practice, be carried out by many methods such as acupuncture, conversation, massage, prayers, diet etc. Healing using character medicine is really an art form, since what is manipulated and changed/developed is really the consciousness and subconscious layers of the patient (the character).

The treatment will re-establish the best version of the patient by upgrading the patient’s character by “balancing in the energies”. Such a balancing cannot be causal, if the original reason to the unbalance is not found; just treating the unbalance, it must be emphasized, is a mere treatment of symptoms and not a healing.

## DISCUSSION

We now have an outline of four systems operating with respectively 2, 3, 4 and 5 elements. We can see these elements as being equally comprehensive and precise depending on the perspective you chose to interpret the world from. One could argue that using 5-ray systems should result in more precise conclusions than a 2-ray system, and from both a mental and a spiritual position this is obviously right. The 10-ray system, like the Jewish Kabbalah, seems to be extremely precise in mapping the conditions of human consciousness. But what is most important in medicine is that the therapist uses a tool that in the most efficient way helps the patient and this will often be founded in the philosophy of the patient. The most efficient system is thus the system that most precisely matches the patient's own worldview. And every perspective will anyway be able to describe the human being as consisting of the elements described.

With a clearly defined system we have an outline of the human character. This makes it possible to plan an efficient treatment for a client, as has been done now for a number of different diseases and health conditions[16-40], which has lead to remarkable clinical results also with patients that could not be cured by standard biomedical treatment[2-6]. By being in touch with the person and by interpreting the character from the chosen perspective, it is possible to evaluate which parts of the character that are balanced and which parts unbalanced – that is, where do we find the qualities of the elements in suitable amounts and where do we find them over or under-represented. For instance can a person in the Chinese 5 element system appear as being consisting of too much water and too less of fire. This means that characteristics as *winter, dead, black and cold,* are particularly visible in the person, while characteristics as *joy, growth, warm and summer* seem particularly under-represented. In this way the goal of the treatment will be to restore the balance by reducing the qualities of water and increasing the qualities of fire. The different systems contain different methods of restoring balance.

None of the elements' characteristics are opposites of each other. For instance a person described from the Yin/Yang system can be too cold in his Yin and too warm in his Yang at the same time. In this case balance will be restored, by adding cold to the warm and warm to the cold.

In relation to the principle of similarity ("what made you ill is what will also cure you"), these systems differ in the way that they seek to balance the energies by *reducing* the symptoms with the help of the symptoms' opposite - that is, to add cold to the warm. Working after the principle of similarity, on the other hand, leads you to *increase* the symptom by adding even more cold to the cold.

In this way we can describe different approaches to treatment. A *balancing* approach, which seeks to reduce the symptom and a *healing* approach, which seeks to increase the symptom and thereby bring the client to an awareness of the causal factor. In case you want to use an approach to 'western nosology and semiotology' it could be necessary to mark the etiological way of homogeneous, heterogeneous, sequential and cascadic types of 'symptoms' to be treated instead to point out only the "causal" factors.

The healing that occurred has recently been understood in greater detail[41-50], and especially Antonovsky's concepts of "sense of coherence" and "salutogenesis"[51-58] has been very useful to understand the remarkable healing powers of the ancient medical systems. Using this concept, it can be argued that holistic medicine, energy medicine, character medicine etc in spite of very different conceptualisations, basically induce the same kind of existential healing as is seen in psychodynamic psychotherapy, which has now been documented to be extremely efficient with a long list of physical, existential, sexual, and psychiatric diseases and health conditions[59-61]; and quite remarkably more efficient that standard treatment[59-61].

The balancing approach in character medicine[12-15] probably also contains a healing effect, since already the establishment of contact between client and therapist and the definition of their mutual relationship creates a space of transference and counter transference, which will take the client back to previous events in life. However, in this approach the therapist will not be working with these processes in a conscious and goal-oriented way. In the spirit of the Swiss psychiatrist, Carl Gustav Jung (1875-1961)[62], the founder of analytical psychology, we prefer a person and individual-oriented way in treatment with a treatment that in the end will make the patient aware of his purpose of life and true inner nature[7-15].
